# Serpin-positive *Bifidobacterium breve* CNCM I-5644 improves intestinal permeability in two models of irritable bowel syndrome

**DOI:** 10.1038/s41598-022-21746-8

**Published:** 2022-11-17

**Authors:** Edgar Torres-Maravilla, Sophie Holowacz, Johanne Delannoy, Loïc Lenoir, Elsa Jacouton, Sandie Gervason, Maëva Meynier, Anne-Sophie Boucard, Frédéric A. Carvalho, Frédéric Barbut, Luis G. Bermúdez-Humarán, Philippe Langella, Anne-Judith Waligora-Dupriet

**Affiliations:** 1 INRAE, AgroParisTech, Micalis Institute, Université Paris-Saclay, 78350 Jouy-en-Josas, France; 2 Université Paris Cité, INSERM, 3PHM, F-75006 Paris, France; 3 PiLeJe Laboratoire, 37 Quai de Grenelle, 75015 Paris Cedex 15, France; 4 INSERM UMR 1107 NeuroDol, University of Clermont Auvergne, 63001 Clermont-Ferrand, France; 5 National Reference Laboratory for C. Difficile, Hôpital Saint-Antoine, Assistance Publique-Hôpitaux de Paris, 75012 Paris, France

**Keywords:** Applied microbiology, Gastrointestinal diseases, Bacterial host response

## Abstract

Probiotic supplementation can help to mitigate the pathogenesis of irritable bowel syndrome (IBS) by reinforcing the intestinal barrier, and reducing both inflammation and proteolytic activity. Here, a combination of in vitro tests was performed on 33 *Bifidobacterium* strains as probiotic candidates for IBS. In addition to the classical tests performed, the detection of the serine protease inhibitor (serpin) enzyme capable of decreasing the high proteolytic activity found in IBS patients was included. Three serpin-positive strains were selected: *Bifidobacterium breve* CNCM I-5644, *Bifidobacterium longum* subsp. *infantis* CNCM I-5645 and *B. longum* CNCM I-5646 for their immunomodulation properties and protection of intestinal epithelial integrity in vitro. Furthermore, we found that *B. breve* CNCM I-5644 strain prevented intestinal hyperpermeability by upregulating *Cingulin* and *Tight Junction Protein* 1 mRNA levels and reducing pro-inflammatory markers. The ability of CNCM I-5644 strain to restore intestinal hyperpermeability (FITC-dextran) was shown in the murine model of low-grade inflammation induced by dinitrobenzene sulfonic acid (DNBS). This effect of this strain was corroborated in a second model of IBS, the neonatal maternal separation model in mice. Altogether, these data suggest that serpin-positive *B. breve* CNCM I-5644 may partially prevent disorders associated with increased barrier permeability such as IBS.

## Introduction

Irritable bowel syndrome (IBS) is a heterogeneous disorder of multifactorial (genetic, physiological, psychosocial and environmental) origin, in which abdominal pain and discomfort are associated with altered defecation and/or bowel habits^1^. The pathogenesis of IBS entails alterations in gastrointestinal motility, post-infectious reactivity, visceral hypersensitivity, gut–brain interactions^2^, food sensitivity, carbohydrate and bile acid malabsorption^3,4^, intestinal inflammation ^5^ and microbiota dysbiosis^6^. The systematic review and meta-analysis by Wang et al.^6^, highlighting the association of dysbiosis and IBS, showed that a deficiency of the commensal *Lactobacillus* and *Bifidobacterium* genera and an overgrowth of potentially pathogenic *Enterobacteriaceae* and *Escherichia coli* populations are frequently observed in IBS^7,8^. Dysbiosis contributes to an increase in intestinal permeability^9^ leading to the passage of both bacteria and antigens through the mucosal layer of the gut^10^. This may be responsible for the activation of mucosal immune responses associated with high proteolytic activity evidenced by increased proteases (i.e. trypsin-like, human neutrophil elastase [HNE], elastase-like and cathepsin G-like and proteinase 3-like) in fecal and tissue samples from a subset of patients with IBS^11–13^. Indeed, some IBS patients may present subtle sub-clinical gastrointestinal inflammatory changes indicating a role for the immune activation in driving symptom onset and chronicity^14^

Modulation of the microbiota through probiotic supplementation can help to mitigate the pathogenesis of IBS by reinforcing the intestinal barrier and reducing gut inflammation^7^. Probiotics (including several strains of the genus *Bifidobacterium*), are considered to exert different health-promoting effects such as limiting pathogen colonization/invasion, influencing gut homeostasis and the immune system through changes in innate and/or adaptive immune responses^15^. Moreover, *Bifidobacterium* strains could counter the proteolytic activity found in IBS patients, through their serine protease inhibitor “serpin”, able to act as an effective inhibitor of HNE and, to a lesser extent, pancreatic elastase^16,17^. Novel insights have revealed the importance of the role of proteases^18^ and their counterparts, the protease inhibitors (i.e. elafin or serpin), as essential components in the restoration of intestinal homeostasis upon disruption^11^. In this study, a combination of in vitro tests was performed to screen potential *Bifidobacterium* probiotic strains before testing the selected strains in in vivo preclinical models of IBS. In addition to the classical tests performed for probiotic characterization (i.e. immunomodulation, barrier gut protection), serpin detection and activity monitoring were included as evaluation criteria. The selected candidates were then tested in two murine models of IBS: the low-grade inflammation induced by dinitrobenzene sulfonic acid (DNBS)^19,20^ and the non-inflammatory neonatal maternal separation (NMS) model ^21^.

## Results

### In vitro screening of bacterial strains: anti-inflammatory properties of potential probiotic strains on TNF-α stimulated human HT-29 cells

**Figure 1:**
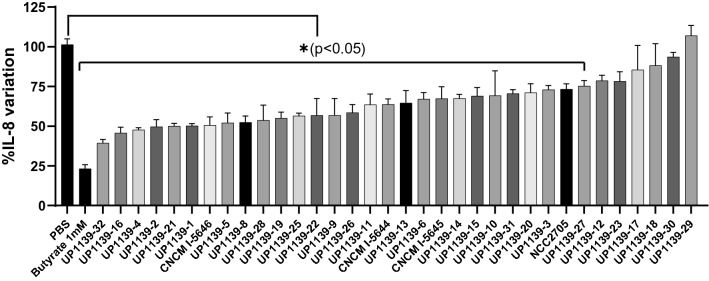
Effect of *Bifidobacterium* strains on IL-8 production by TNF-α stimulated HT-29 cells. IL-8 production was stimulated by incubating HT-29 cells with 5 ng of TNF-α and co-incubation with *Bifidobacterium* strains for 6 h. Results are presented as mean +/− SEM. Comparison between treated groups and the PBS control group by a one-way ANOVA test followed by the Dunnet’s test. *p < 0.05 compared to PBS control group.

Since interleukin (IL)-8 is considered a major inflammatory mediator candidate strains able to inhibit or significantly (*p* < 0.05) reduce IL-8 production can be considered to display anti-inflammatory properties. Twenty-eight (including the NCC2705 reference strain) of the 33 bacterial strains tested significantly (*p* < 0.05) reduced IL-8 production (Fig. 1). *Bifidobacterium longum* UP1139-32 and *B. breve* UP1139-16 reduced IL-8 production by almost 60%, which was the greatest reduction observed; similar reductions were achieved with butyrate used as a positive control. Significant anti-inflammatory effects were observed for thirteen strains of the *B. breve* species (including CNCM I-5644), 9 strains of *B. longum* (including CNCM I-5646 and *B. longum* subsp. *infantis* CNCM I-5645), 4 strains of *B. bifidum*, and 2 strains of *B. adolescentis*. No species-dependent effect was observed.

### Immunomodulatory properties of probiotic strains on human PBMCs

**Figure 2:**
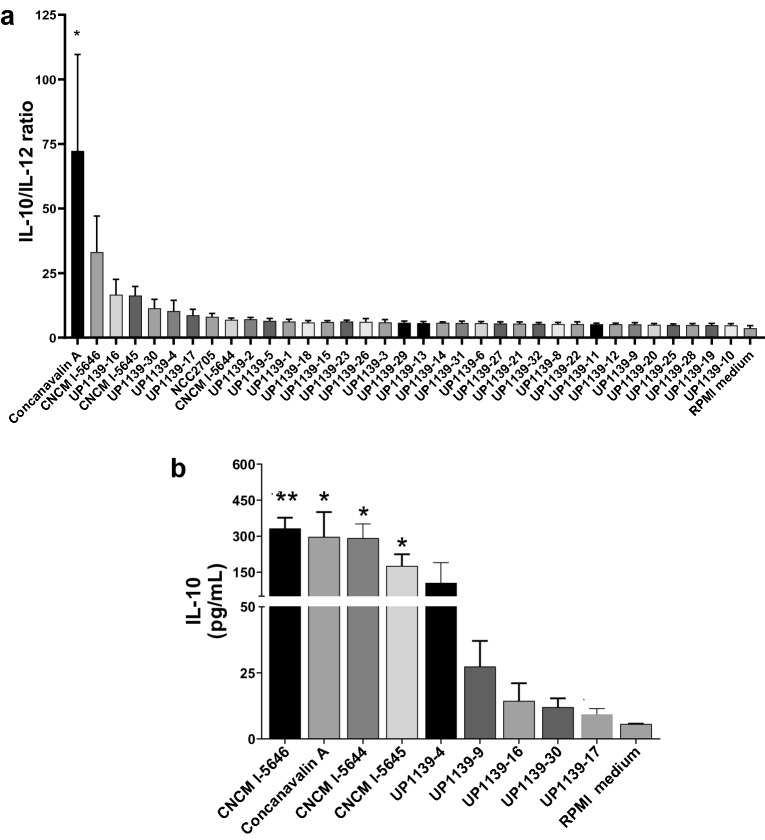
Immunomodulatory properties of probiotic strains on human PBMCs from five donors. (**a**) IL-10/IL-12 ratio after incubation of strains with PBMCs. (**b**) IL-10 immunomodulation after incubation of strains with PBMCs. Cytokines were quantified by ELISA after co-incubation of bacteria and PBMCs from five donors for 48 h. Data are presented as mean (n = 15) ± SEM. A one-way ANOVA test was performed followed by a Dunnet’s test to compare treated groups with RPMI medium group. *p < 0.05, **p < 0.001.

The immunomodulatory properties were evaluated through the IL-12/IL-10 ratio following co-incubation of bacteria with human peripheral blood mononuclear cells (PBMCs) (Fig. 2). As shown in Fig. 2a, 9 strains (UP1139-2, UP1139-4, UP1139-16, UP1139-17, UP1139-30, CNCM I-5644, CNCM-I-5645, CNCM I-5646, NCC2705 included) tended to increase the IL-10/IL-12 ratio with respect to the cut-off value of RMPI medium; in particularly *B. longum* strain CNCM I-5646. However, only the ratio observed for concanavalin-A was significantly higher than RPMI medium (*p* < 0.05). Regarding IL-10 immunomodulation (Fig. 2b), only CNCM I-5644, CNCM I-5645, and CNCM I-5646 strains induced a significantly higher IL-10 concentration (~ 300 pg/mL) that was significantly higher than RPMI medium and in a similar range as concanavalin-A. Basal IL-10 levels were found for other bacterial strains.

### Gut permeability protection abilities of bacterial strains measured by trans-epithelial electrical resistance (TEER) assay in TNF-α stimulated Caco-2 cells

**Figure 3:**
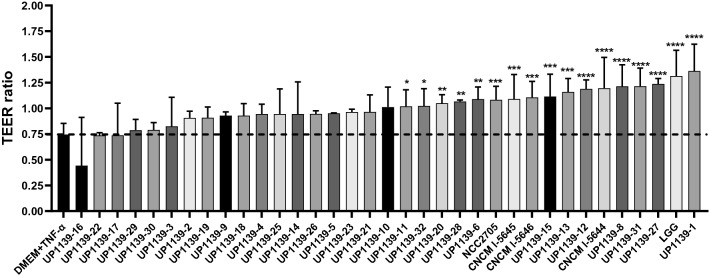
Protective effect of *Bifidobacterium* serpin-positive strains on intestinal barrier integrity measured by Trans-Epithelial Electrical Resistance (TEER). TEER was measured before adding the tested strain in the apical surface of Caco-2 cells for 3 h prior to treatment of the basolateral medium with TNF-⍺ 100 ng/mL for 21 h at 37 °C. Results are presented as mean +/− SEM. *Indicates significant differences between treated groups and DMEM + TNF-α control group. *p < 0.05, **p < 0.01, ***p < 0.001, ****p < 0.0001.

TNF-α induced barrier alteration in Caco-2 cells, decreasing the TEER ratio to a value of 0.74 (Fig. 3). Fifteen *Bifidobacterium* strains significantly improved TEER ratios (*p* < 0.05), highlighting their potential to protect epithelial barrier from TNF-α-induced alteration. The limit of protection was observed with *B. breve* UP1139-11 with a TEER ratio of 1.0 using the one-way ANOVA followed by the Dunnet’s test to compare bacteria and DMEM + TNF-α groups. The maximum TEER value was reached when Caco-2 cells were co-incubated with *B. adolescentis* UP1139-1 strain, with a TEER ratio of 1.36. Remarkably, the 15 strains were serpin-positive strains and belonged to the *B. breve* (7 strains), *B. longum* (6 strains), and *B. adolescentis* species and *B. longum* subsp. *infantis* (1 strain each). The improvements in the TEER ratio observed with these strains were similar to those observed with the reference strain LGG.

### Detection and upregulation of serpin and selection of *Bifidobacterium* strains

**Figure 4:**
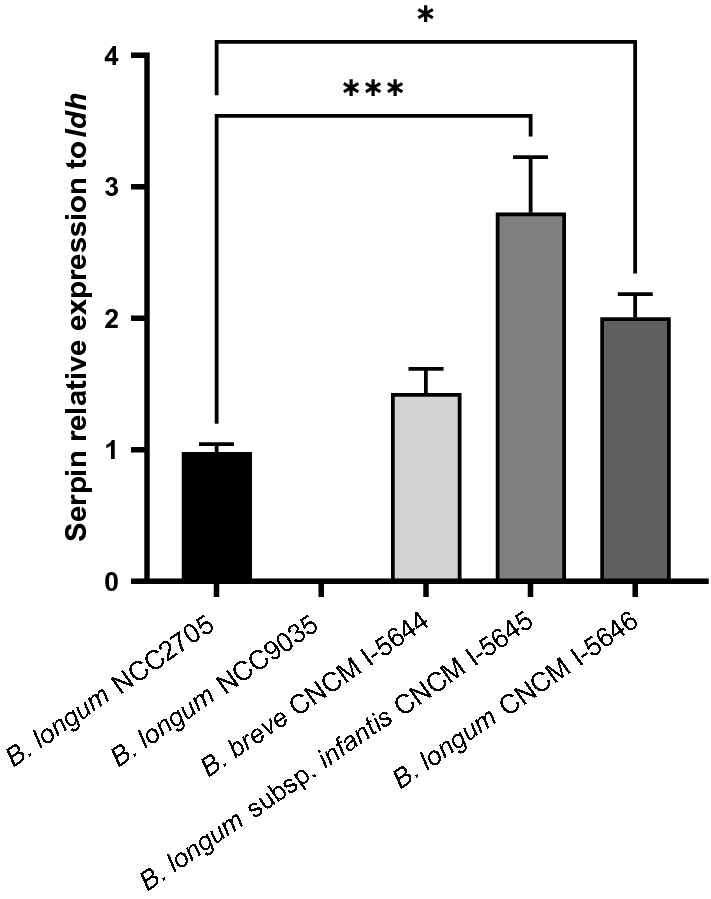
Serpin mRNA levels were quantified in three *Bifidobacterium* strains. The 2^−ΔΔCT^method was carried out to quantify relative gene expression. The lactate dehydrogenase gene was used as a housekeeping gene, *B. longum* NCC2705 as a serpin-positive reference bacteria and *B. longum* NCC9035 as a negative control. Results are presented as mean +/− SEM. **p < 0.05, ***p < 0.001 compared to NCC2705 group.

The gene coding for serpin was detected by PCR in 21 out of the 33 strains (Table 1). Most of the positive strains belonged to the *B. longum* species (11 strains), followed by the *B. breve* species (9 strains), and the *B. longum* subsp. *infantis* (1 strain). No PCR amplification was found for *B. adolescentis* and *B. bifidum* species. Three serpin-positive strains of different *Bifidobacterium* species showed high performance in all three in vitro assays. *B. breve* CNCM I-5644, *B. longum* subsp. *infantis* CNCM I-5645 and *B. longum* CNCM I-5646 all showed anti-inflammatory properties in TNF-⍺-stimulated HT-29 cells, immunomodulatory properties with the induction of a high IL-10/IL-12 ratio and/or a strong stimulation of IL-10 production by PBMCs and the ability to protect the epithelial barrier (see Supplementary Fig. S2 online). To confirm that these strains can express their *serpin* gene (and not only harbor the gene), we quantified the levels of serpin-encoding mRNA by qPCR (Fig. 4). Expression of *serpin* gene in *B. breve* CNCM I-5644 was similar to that of the reference strain NCC2705. In contrast, *B. longum* subsp. *infantis* CNCM I-5645 and *B. longum* CNCM I-5646 expressed significantly 2.8- and 2.4-fold more times *serpin* gene than NCC2705, respectively (*p* < 0.05). These three strains were then chosen for in vivo experimentation.

**Table 1:** *Bifidobacterium* strains used.

Organism	Collection no	Origin	Serpin detection	Organism	Collection no	Origin	Serpin detection
*B. adolescentis*	UP1139-1	Human		*B. breve*	UP1139-18	Human	+
*B. adolescentis*	UP1139-2	Human		*B. breve*	UP1139-19	Human	+
*B. bifidum*	UP1139-3	Human		*B. breve*	UP1139-20	Human	
*B. bifidum*	UP1139-4	Human		*B. breve*	UP1139-21	Human	+
*B. breve*	UP1139-5	Human		*B. longum*	UP1139-22	Human	+
*B. breve*	UP1139-6	Human		*B. longum*	UP1139-23	Human	+
*B. breve*	CNCM I-5644	Human	+	*B. longum*	CNCM I-5646	Human	+
*B. breve*	UP1139-8	Human	+	*B. longum*	UP1139-25	Human	+
*B. breve*	UP1139-9	Human	+	*B. longum*	UP1139-26	Human	+
*B. breve*	UP1139-10	Human		*B. longum*	UP1139-27	Human	+
*B. breve*	UP1139-11	Human	+	*B. longum*	UP1139-28	Human	+
*B. breve*	UP1139-12	Human	+	*B. longum*	UP1139-29	Human	+
*B. breve*	UP1139-13	Human		*B. longum*	UP1139-30	Human	+
*B. breve*	UP1139-14	Human		*B. longum*	UP1139-31	Human	+
*B. breve*	UP1139-15	Human		*B. longum*	UP1139-32	Human	+
*B. breve*	UP1139-16	Human		*B. longum* subsp. *infantis*	CNCM I-5645	Human	+
*B. breve*	UP1139-17	Human	+				

^**+**^positive PCR-detection for serpin.

### Administration of *Bifidobacterium* strains prevents the development of lesions and low-grade inflammation

**Figure 5:**
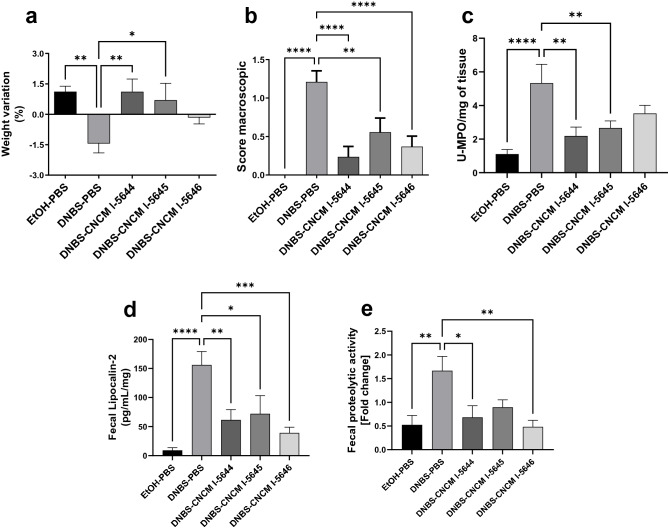
Effect of *Bifidobacterium* strains in the DNBS low-grade inflammation murine model. (**a**) Body weight variation, (**b**) Macroscopic score, (**c**) MPO/mg of tissue, (**d**) Fecal Lipocalin-2 (Lcn-2) and (**e**) Proteolytic activity. Results are presented as means ± SEM. Results of with a one-way ANOVA test followed by the Dunnet’s test comparing the DNBS-PBS group to others groups. *p < 0.05, **p < 0.01, ***p < 0.001, ****p < 0.0001.

To investigate the characteristics of the candidate probiotic strains in the context of IBS, we experimentally induced the chronic low-grade inflammation and gut dysfunction in mice by administration of two episodes of DNBS instillations. In this model, symptoms are inconspicuous. However, as shown in Fig. 5a, mice that received only DNBS showed a significant weight loss (*p* < 0.05) compared to the PBS group, which gained weight during the experiment. Administration of *B. breve* CNCM I-5644 and *B. longum* subsp. *infantis* CNCM I-5645 significantly prevented DNBS-induced weight curve breakdown (*p* < 0.05), with a weight gain of 0.8% and 1%, respectively; this weight gain was not observed with *B. longum* CNCM I-5646 (Fig. 5a). DNBS induced hyperemia, i.e., presence of adhesions between the colon and other intra-abdominal organs and changed the consistency of the feces (diarrhea) (Fig. 5b). The low-grade inflammation induced by DNBS was reflected by the increase in neutrophilic infiltration (5 U MPO/mg increase in colonic tissues; Fig. 5c), the increase in Lipocalin-2 (Lcn-2) concentration (a marker of early inflammation) (Fig. 5d) and the increase in proteolytic activity in the feces (Fig. 5e) of the DNBS control group compared to the EtOH-PBS group (*p* < 0.05). Globally, the oral administration of *Bifidobacterium* strains prevented the DNBS-induced macroscopic damage in colon samples. *B. breve* CNCM I-5644 and *B. longum* subsp. *infantis* CNCM I-5645 significantly reduced MPO to basal levels (1 U MPO; *p* < 0.05; Fig. 5c). As expected, both Lcn-2 concentration (Fig. 5d) and proteolytic activity (Fig. 5e) in the feces were reduced by oral administration of *B. breve* CNCM I-5644 and *B. longum* CNCM I-5646 strains at concentrations similar to those of the untreated group (EtOH-PBS).

**Figure 6:**
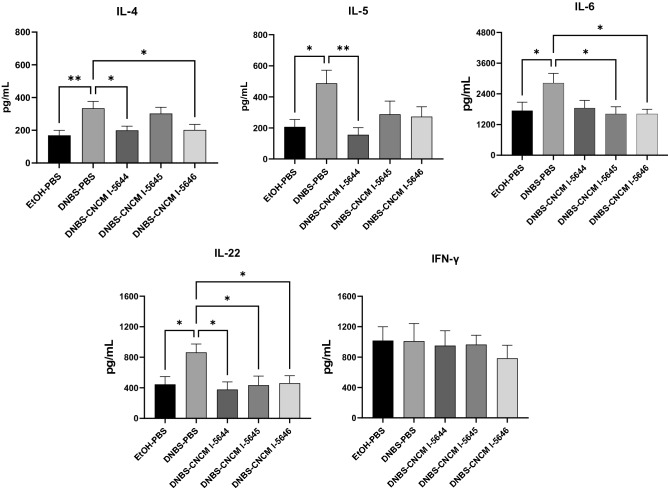
Impact of *Bifidobacterium* strains on systemic T-helper balance. Cytokines secreted by splenocytes from each group of mice, stimulated for 48 h with CD3/CD28, were measured using ELISA. Results of non-parametric Mann–Whitney test comparing the DNBS-PBS group to others groups. *p < 0.05, **p < 0.01.

Cytokines secreted by lymphocytes from splenocytes showed a Th1 bias of the systemic immune system in DNBS-treated mice (Fig. 6). Indeed, in the DNBS-PBS group, we observed a significant increase in the pro-inflammatory cytokines IL-4, IL-5, IL-6, IL-22 (*p* < 0.05), but not for INF-γ compared to the EtOH-PBS group. Oral administration of the three *Bifidobacterium* strains tested tended to decrease the pro-inflammatory cytokines. In particular, *B. breve* CNCM I-5644 reduced IL-4, IL-5 and IL-22 and tended to reduce IL-6 compared to the DNBS control group (Fig. 6).

### Administration of *Bifidobacterium* strains prevents barrier disruption in the murine model of low-grade inflammation induced by DNBS

**Figure 7:**
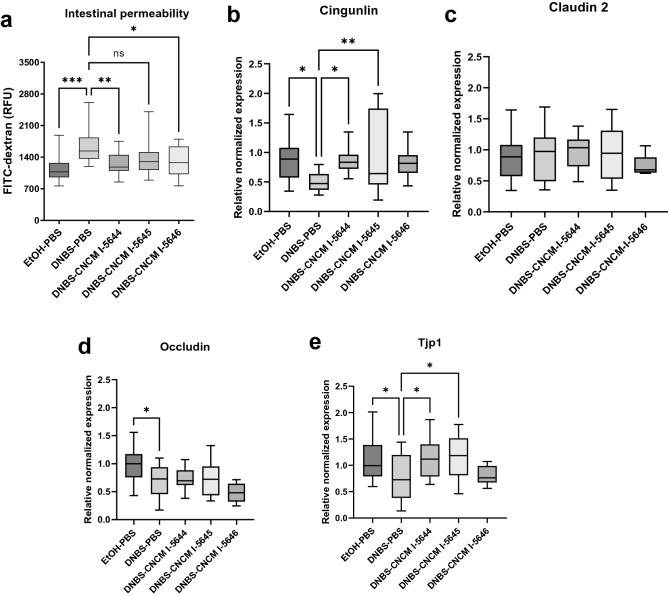
Regulation of intestinal tight junction proteins in the DNBS low-grade inflammation model. (**a**) Intestinal permeability measured by FITC-dextran, and colonic expression of (**b**) *Cingulin*, (**c**) *Claudin 2*, (**d**)* Occludin*, and (**e**) *Tjp1* in each mice group. The 2^−ΔΔCT^method was carried out to quantify relative gene expression. The *Rpl19* and *Tbp* genes was used as a housekeeping gene. Results of a one-way ANOVA test followed by the Dunnet’ test comparing the DNBS-PBS group to others groups. *p < 0.05, **p < 0.01, ***p < 0.001.

Particular attention was given to intestinal permeability, the increase of which is considered to play a key role in IBS. Mice were administered FITC-dextran to assess intestinal permeability. The DNBS group showed a significant increase of 1639 Relative Fluorescence Units (RFUs of FITC-dextran) compared to the EtOH-PBS group with 1142 RFUs (*p* < 0.05; Fig. 7a). *Bifidobacterium breve* CNCM I-5644 and *B. longum* CNCM I-5646 significantly decreased RFUs by 22–25% (*p* < 0.05), thus, reducing intestinal hyperpermeability. To better understand how bacteria affects intestinal permeability in mice, the expression of cell junction complex proteins was measured by qPCR (Fig. 7b–e). mRNA levels of *Cingunlin, Occludin* and *Tjp1* were significantly downregulated in the DNBS control group (*p* < 0.05; Fig. 7b,d,e), whereas *Claudin* 2 was not affected (Fig. 7c). *Bifidobacterium breve* CNCM I-5644 and *B. longum* subsp. *infantis* CNCM I-5645 significantly restored the *Cingunlin* and *Tjp1* mRNA transcripts to similar levels as in the EtOH-PBS control group (onefold change). No significant changes were observed with *B. longum* CNCM I-5646.

### Impact of *B. breve* CNCM I-5644 on the NMS model

**Figure 8:**
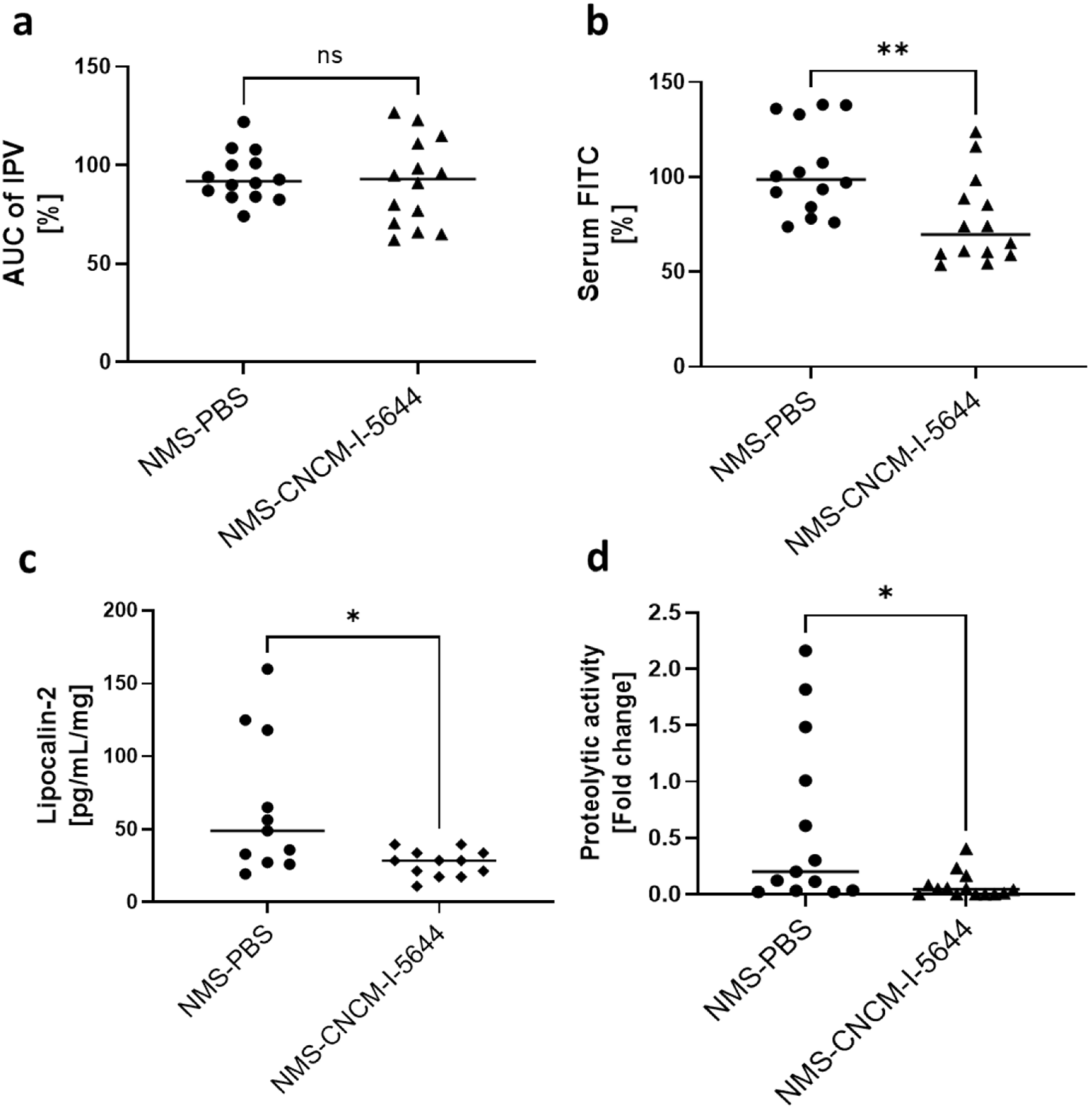
Impact of oral administration of *B. breve* CNCM I-5644 in the non-inflammatory NMS murine model. (**a**) Colonic hypersensitivity, measured as the area under the curve (AUC) of intracolonic pressure variation (IPV), was calculated by trapeze method between 60 and 100 mmHg for each group of mice. (**b**) Intestinal permeability measured by FITC, (**c**) and Lipocalin-2 and (**d**) proteolytic activity in feces. Comparisons were performed by a one-way ANOVA test followed by the Dunnett’s test. Results are presented as mean +/− SEM (n = 14). *p < 0.05, **p < 0.01 compared to NMS-PBS group.

Considering the overall results obtained in the DNBS low-grade inflammation model with *B. breve* CNCM I-5644, especially its ability to restore barrier function, we decided to test this promising strain in the NMS model, which mimics symptoms of IBS induced by stress (Fig. 8). NMS-induced stress in mice (NMS-PBS group) let to a significant increase in Intracolonic pressure variation (IPV) to CRD at the highest distension volumes, reflecting colonic hypersensitivity (see Supplementary Fig. S3 online). No effect of *B. breve* CNCM I-5644 was observed on this parameter (Fig. 8a). In contrast, the strain was able to restore barrier function, as shown by a significant 25% decrease in FITC-dextran compared to the NMS-PBS group (*p* < 0.01; Fig. 8b). Nevertheless, no significant difference was observed in the expression of cell junction complex proteins (see Supplementary Fig. S4 online). Finally, oral administration of *B. breve* CNCM I-5644 significantly reduced Lcn-2 levels by 60% (*p* < 0.05; Fig. 8c), and proteolytic activity in the feces by 85% (*p* < 0.05; Fig. 8d).

## Discussion

In this study, we applied a set of in vitro screenings that allowed us to identify a promising candidate probiotic strain to improve intestinal permeability in two different murine models which overlap with symptoms of IBS. In addition to the widely used as readout for in vitro probiotic characterization, such as immunomodulatory (IL-10/IL-12 ratio and IL-8 levels) and barrier protection (TEER values) properties, we decided to include PCR detection of the gene coding the serine protease inhibitor (serpin). Serpin was first identified in *Bifidobacterium longum* species^22^. Serpin anti-proteases could improve intestinal homeostasis by counteracting the elevated proteolytic activity often found in IBS patients^23^, thus becoming an interesting strategy to use for the selection of suitable probiotics for IBS. Some patients with IBS had been found with low-grade inflammation, more likely those to suffer diarrhea-predominant IBS (IBS-D) or post-infectious IBS (PI-IBS), than other IBS subtypes^24^ as observed by increased lamina propria immune cells in the colonic mucosa^25,26^, as well as increased levels of pro-inflammatory cytokines including IL-1β, TNF-α, IL-6 and IL-8, and reduced levels of anti-inflammatory cytokines such as IL-10^27^. Nowadays, it is unclear whether serpin-positive strains play a direct role on the anti-inflammatory response, but in previous studies, other protease inhibitors such as elafin^28^ and synthetic protease (UAMC-00050) had immunomodulatory effects as a consequence of their protease inhibition activity^29^. Here, almost all *Bifidobacterium* serpin-positive strains tested showed anti-inflammatory properties by inhibiting the production of IL-8 in the HT-29 cells/TNF-α model. Serpin can also be considered as another bacterial compound with previously reported anti-inflammatory properties, such as compounds from subcellular fractions (cytoplasm, surface exopolysaccharides [EPS])^30^ and metabolites (i.e. short chain fatty acids [SCFAs]: butyrate, propionate, acetate)^31^. Furthermore, although our results did not show a direct correlation between serpin-positive strains and IL-10/IL-12 ratios, serpin-positive strains (CNCM I-5644, CNCM I-5645, CNCM I-5646 and UP1139-9) induced the highest IL-10 concentrations in human PBMCs. The key role of IL-10 in the control of intestinal inflammation has been demonstrated in IL-10 knockout mice^32^, as well as by the oral administration of a recombinant strain of *B. bifidum* BS42 efficiently delivering in situ this cytokine in a murine model of low-grade intestinal inflammation^33^. Serpin-positive *Bifidobacterium* strains reinforced the intestinal barrier (reduced paracellular values of TEER in Caco-2 cells in vitro). This could be explained by anti-protease activity that could lead to the neutralization of the altered proteolytic enzymes produced in the intestinal lamina propria that have a harmful effect on the intestinal barrier^34^. Increased intestinal membrane permeability is found in IBS patients^35,36^, this symptom was induced in the DNBS low-grade inflammation model, in which the three selected strains tended to reinforce the intestinal barrier in mice as evidenced by decreased FITC levels. In particular, *B. breve* strain CNCM I-5644 reinforced the intestinal barrier in both IBS in vivo models. It is known that *Bifidobacterium* strains can improve the physical barrier by increasing the production of mucus-covered epithelium through the secretion of metabolites such as SCFAs^37^ or gamma aminobutyric acid (GABA) neurotransmitter that upregulate the major mucin *MUC2* and modulate goblet cell function by reducin*g* FITC-dextran tracers^38,39^. Here, we demonstrated that CNCM I-5644 and CNCM I-5645 upregulate the gene encoding *Cingulin* involved in the formation of a tight junction creating a paracellular barrier in epithelial and endothelial cells protecting them from the external environment^40,41^, and upregulate *Tjp1* that encodes *ZO-1*. Tight junctions upregulation by *B. longum* is associated with signaling through TLR2, the major pattern recognition receptor for Gram-positive bacteria^42^. Taken together, our results are consistent with other studies on *Bifidobacterium* species^39,43,44^. Pro-inflammatory cytokines also contribute to alterations in gut permeability: IL-4 induces epithelial hypertrophy, IL-5 promotes eosinophil cellular infiltration and IL-6 modulates the excitability of submucosal neurons and increases colonic secretory function^45^. All of these cytokines were reduced in DNBS-treated mice that received CNCM I-5644. The three bacterial strains were able to reduce MPO and fecal Lipocalin-2, two vital markers of neutrophil infiltration, to combat different types of microbial activities^46^.

Dysregulated proteolysis elicits structural and functional changes in the mucosal barrier and participates in inflammation^34^, with proteolytic activity usually increased in IBS patients with diarrhea-predominant^47^, as observed in the two in vivo murine models used in this work. For this reason, the use of a protease inhibitor like camostat mesilate (CM) has been suggested to treat some IBS subtypes^48,49^. *Bifidobacterium longum* NCC2705 serpin-positive strain has previously been successfully tested in gliadin-induced immunopathology in NOD/DQ8 mice, where serpin was shown to be essential for the beneficial effect of the strain^16^. In our study, we confirmed that all three strains, CNCM I-5644, CNCM I-5645, and CNCM I-5646, expressed the serine protease inhibitor “serpin” at similar expression levels to that observed with the reference strain *B. longum* NCC2705, confirming the interest of using serpin as a criterion for probiotic selection for IBS.

As the DNBS model explore intestinal permeability and low-grade inflammation, the latter of which is observed only in a number of IBS patients, in particular those with post-infectious IBS (PI-IBS) or diarrhea-predominant IBS (IBS-D) but not in all of them^24,50^, a second non-inflammatory model of IBS was used. We choose the NMS model, to test the most performant strain in the DNBS low-grade inflammation model, i.e. *B. breve* CNCM I-5644. Early life stressors, such as maternal deprivation, can increase the risk of developing IBS in adulthood^51^ and induce a shift of the gut microbiota towards dysbiosis^52^. Additionally to increased intestinal permeability, a severe and recurrent pain (colonic sensibility) is observed in IBS patients^35^. The NMS model is characterized by a disruption of intestinal permeability and colonic sensibility due to increase in basal corticosterone level generated by psychological stress^53,54^. Here, *B. breve* CNCM I-5644 showed gut intestinal protection, as evidenced by reduced FITC-dextran tracer and decreased fecal proteolytic activity; it did not change susceptibility to stress in the colorectal distension (CRD) test. However, the addition of CNCM I-5644 with another strain (synergistic effect) could improve the latter. According to Ait-Belgnaoui et al. ^55^, the oral administration of *B. longum* or *L. helveticus* strains separately was less effective in reducing visceral pain in stressed mice than their combination. The paracellular space is sealed by the tight junction, which is maintained by a complex network of protein interactions^56^. In our study, no significant regulation of tight junction protein genes was found in colon samples; this could be explained by the expression and redistribution of tight junction proteins^57^, region-specific reduction during stress alteration, and rapid turnover and trafficking of tight junction proteins^58^.

In conclusion, this study highlights a wide range of immunomodulatory properties and beneficial effects on the host intestinal barrier function of several *Bifidobacterium* strains, including serpin-positive *B. breve* CNCM I-5644, and provides further evidence of their efficacy to alleviate and protect the intestinal barrier in two models of IBS. These data suggest that serpin-positive *B. breve* CNCM I-5644 in particular, may be effective in preventing disorders associated with increased barrier permeability such as IBS. It could be interesting to evaluate the synergistic interaction of *B. breve* CNCM I-5644 with other probiotic strains to improve colonic hypersensitivity. This work also demonstrates the importance of selecting the proper in vitro experiments, such as readouts used in this study: IL-8 and IL-10 immunomodulation, intestinal barrier protection and serpin detection, three important probiotic characteristics for the selection of new candidate probiotic strains for the restoration of intestinal homeostasis in the context of IBS.

## Methods

### Strains

Thirty-three *Bifidobacterium* strains from the collection of UMRS-1139 Université de Paris were screened (Table 1). To validate our in vitro studies, the serpin-positive *B. longum* strain NCC2705 and their derivate serpin-negative NCC9035 were used as reference^16^. All bacteria were grown in Man-Rogosa-Sharpe (MRS) medium (Difco, France), supplemented with 0.5 mg/mL cysteine (Sigma-Aldrich) at 37 °C in an anaerobic chamber. Strains were grown as previously described by Neau et al.^59^. Briefly, bacteria were collected 1 h after the beginning of the stationary phase, harvested by centrifugation, washed twice, re-suspended in phosphate-buffered saline (PBS; Gibco, Thermo Scientific, Illkirch, France) containing 15% glycerol, and stored at − 80 °C until further assays. Colony forming units (CFU) were estimated by serial dilution on agar plates. Three independent growth experiments were performed for each strain. Preparation of bacterial inoculum for administration in mice was performed as described above and lyophilized.

### Experiments with the HT-29 cell line

We obtained the human colon adenocarcinoma cell line HT-29 from the European Collection of Authenticated Cell Cultures (ECACC; Sigma). HT-29 cells were seeded at 5 × 10^4^ cells/mL per well in Dulbecco's modified Eagle's medium (DMEM) supplemented with 1% glutamine (Lonza, Switzerland), 10% heat-inactivated fetal bovine serum (FBS, Eurobio), 1% penicillin–streptomycin (Lonza, Switzerland) at 37 °C/10% CO_2_ air atmosphere. The medium was changed every 2 days. After confluence was reached, medium was changed with 5% (v/v) FBS, 1% glutamine and 0.1% penicillin–streptomycin and TNF-α (5 ng/mL, Peprotech, NJ) to which we added bacteria (at multiplicity objet infection [MOI] of 40-ratio of bacteria to eukaryotic cells), PBS glycerol was used as control^60^. After co-incubation of 6 h, cell supernatants were collected and frozen at − 80 °C until subsequent analysis of IL-8 concentrations by enzyme-linked immunosorbent assay (ELISA) (Biolegend, San Diego, CA) according to the manufacturer’s instructions.

### Experiments with peripheral blood mononuclear cells

Human peripheral blood mononuclear cells (PBMCs) were isolated from the qualified buffy coat cells of five healthy donors (all male; aged < 65 years, with a body mass index < 30; negative for HIV and hepatitis A and B viruses, donors provided written informed consent), which were provided by Etablissement Français du Sang (EFS), experiments were performed in accordance with the relevant guidelines and regulations of EFS with the following agreement: CPSL UNT_18/EFS/030. PBMCs were adjusted and seeded in were seeded in 48-well plates at a density of 6.75 × 10^5^ cells/well in Iscove’s modified Dulbecco’s medium (Sigma-Aldrich, France) supplemented with 10% fetal bovine serum (FBS; Gibco, Thermo Scientific, Illkirch, France), 1% l-glutamine, 0.1% penicillin–streptomycin, and 0.1% gentamicin^59^. Bacteria from three independent cultures were added at a MOI of 1:10 in 50 μL of PBS. Plates were incubated for 48 h at 37 °C in 5% CO_2_. Samples were finally stored at − 80 °C until further analysis of IL-10 and IL-12p70 concentrations by ELISA-Multiplex (Biorad, France).

### In vitro permeability assay in Caco-2 cells

We obtained the Caco-2 cell line from the American Type Tissue Collection (ATCC), and maintained it in DMEM supplemented with glutaMAX™ (Lonza, Switzerland), 20% heat-inactivated FBS, and 1% non-essential amino acids (Gibco, Thermo Scientific, Illkirch, France). Caco-2 cells were grown on Transwell semi-permeable filter support (12 mm-diameter wells, polystyrene membranes with 0.4 μm pores, Costar, Corning) and seeded at 2.9 × 10^4^ cells per well with Trans-Epithelial Electrical Resistance (TEER) readings of > 1500 ohms cm^−2^ (8–10 days, HTS robot REMS autosampler, World precision instruments). TEER was measured before adding 1 × 10^6^ CFU of each *Bifidobacterium* strain onto the apical surface and co-incubated for 3 h at 37 °C/10% CO_2_, prior to treatment of the basolateral medium with TNF-⍺ (100 ng/mL) for an additional 21 h. Experiments were performed at least in duplicate. The strain *Lactobacillus rhamnosus* LGG was used as positive control^61,62^. The resulting data presented as a TEER ratio:

$$TEER\;ratio = \frac{{{{TEER\;treatment\;T24} \mathord{\left/ {\vphantom {{TEER\;treatment\;T24} {TEER\;treatment\;T0}}} \right. \kern-\nulldelimiterspace} {TEER\;treatment\;T0}}}}{{{{TEER\;control\;T24} \mathord{\left/ {\vphantom {{TEER\;control\;T24} {TEER\;control\;T0}}} \right. \kern-\nulldelimiterspace} {TEER\;control\;T0}}}}$$

### PCR-detection of *serpin* gene

Bacterial DNA extractions were performed according to the procedures of the Instagene kit (BIORAD, France). Primers with specificity for *B. longum* serpin and its derivatives were obtained targeting the *BL0108 serpin* gene insertion sequence (Genback number accession: AAN23973.1). Primers were as follows: forward, 5′-(TCGATGGTGAATCGCGGTAG)-3′, and reverse, 5′-(TATGTTCAAGCCGAAGGCA)-3′. Each PCR was performed in a thermocycler (ThermoFisher, France) with the following program: initial denaturation step of 3 min at 95 °C, followed by 35 cycles of denaturation for 30 s at 95 °C, annealing for 30 s at 55 °C, and extension for 1 min at 72 °C and then elongation for 5 min at 72 °C. The detection was confirmed using agarose gel electrophoresis.

### Serpin gene expression

Cell pellets from CNCM I-5644, CNCM I-5645, CNCM I-5646 and *Bifidobacterium longum* strain NCC2705 (reference strain^16^) were harvested 1 h after the beginning of the stationary phase by centrifugation. Total RNA was extracted from bacterial using an RNeasy minikit (Qiagen) with additional DNase treatment. One microgram of total RNA was reverse transcribed using an Applied Biosystems High-Capacity cDNA Reverse Transcription Kit (TermoFisher, France). Real-time quantitative PCR (qPCR) was performed using diluted (100-fold) cDNA duplicates and a StepOnePlus System (Applied Biosystems, France) according to conditions previously reported by McCarville, et al.^16^.

### Low-grade inflammation model and bacteria administration

Specific pathogen-free male C57BL/6 mice (6–8 weeks old) (Janvier, Le Genest Saint Isle, France) were maintained under normal breeding conditions in the animal care facilities of Infectiologie Expérimentale des Rongeurs et des Poissons (IERP) of Institut National de Recherche pour l’Agriculture, l’Alimentation et l’Environnement (INRAE, Jouy-en-Josas, France). They were housed in cages of five. Our experiments were performed in accordance with European Union legislation on animal welfare and were approved by Comité d’Éthique en expérimentation animale (COMETHEA), our local committee on animal experimentation (n°16744-201807061805486 v2) and in compliance with the Animal Research: Reporting of In Vivo Experiments (ARRIVE) relevant guidelines.

Low-grade inflammation was induced by DNBS according to the protocol of Martín et al.^19^ with small modifications (see Supplementary Fig. S1 online). Briefly, mice were anesthetized by an intraperitoneal (i.p.) injection of 0.1% ketamine (Imalgene 1000, Merial, France) and 0.06% xylazine (Rompun, Bayer, France). Inflammation was triggered by intra-rectal injection of 200 mg kg^−1^ of DNBS (ICN Biomedical Inc., Santa Ana, CA) re-suspended in 30% ethanol-PBS (DNBS-PBS) (day 1). Mice in the non-inflamed group received 30% EtOH-PBS alone. Ten days after DNBS injection (day 14) , 200 µL of PBS solution containing 1 × 10^9^ CFU of the strain tested, or 200 µL of PBS, were intragastrically administered every day for 10 days (gavage period). Low-grade inflammation was reactivated 21 days after the first DNBS injection (recovery period) with a second injection of 100 mg kg^−1^ of DNBS solution (Day 21). During this reactivation period, mice lost weight until the end-point of the experiment (day 24). The study groups were control non-inflamed group (EtOH-PBS), control inflamed group (DNBS-PBS), *B. breve* CNCM I-5644 inflamed group (DNBS-CNCM I-5644), *B. longum* subsp. *infantis* CNCM I-5645 inflamed group (DNBS-CNCM I-5645) and *B. longum* CNCM I-5646 inflamed group (DNBS-CNCM I-5646). After euthanasia, the colon and small intestine were removed, opened longitudinally and immediately assessed for visible damage, macroscopic scores, and myeloperoxidase (MPO) activity levels and Intestinal permeability integrity by using fluorescein isothiocyanate-dextran (FITC-Dextran) were characterized as previously described^19,63^. We quantified inflammation by assessing the levels of the pro-inflammatory cytokines IL-4, IL-5, IL-6, IL-22 and IFN-γ from spleen by enzyme-linked immunosorbent assay (ELISA, Biolegend, California, U.S.). For Lcn-2, feces samples were homogenized (50 mg/mL) in reaction buffer (0.5% W/V NaHCO_3_, pH 8.3) and ceramic beads (diameter: 1.4 and 2.8 mm) using a Precellys tissue homogenizer in cold conditions. Samples were centrifuged for 20 min, and the supernatant was frozen at − 80 °C. Lipocalin-2 (Lcn-2) was assayed using Duoset murine Lcn-2 ELISA kit (R&D Systems, Minneapolis, MN). Fecal protease activity was determinated photometrically by using azocasein as a proteolytic substrate^64^.

### Intestinal tight junction proteins

Total RNA was isolated from 20 to 30 mg of colon samples with a RNeasy Mini Kit (Qiagen). DNAse column treatment was used to eliminate potential DNA contamination. RNA quantity and quality were confirmed with a NanoDrop apparatus (Thermo Scientific) and by agarose gel electrophoresis. Only samples with intact RNA were used for subsequent cDNA synthesis with iScript reverse transcriptase (Bio-Rad, France): 1000 μg of the total RNA preparation was used for each sample. Quantitative real-time PCR (qPCR) was performed with diluted cDNA (10x) in triplicate and with an iQ5 Real-Time Detection System (Bio-Rad, France), and according to conditions previously reported by Martín et al.^20^. The reaction mixture consisted of Sofast Evagreen Supermix (Bio-Rad), primers at 0.5 μM, and 2 μL of diluted cDNA. Values are expressed as relative fold differences normalized to the housekeeping gene, Ribosomal protein L19 (*Rpl19*) and TATA box binding protein (*Tbp*) by the 2^−ΔΔCT^method. All procedures were performed according to the manufacturers’ instructions. The entire list of primers used can be found as Supplementary Table S1 online.

### Assessing effects on NMS model

C57Bl/6J pregnant mice were purchased from Janvier laboratories (Le Genest Saint Isle, France) and singe-housed, up to birth of the pups. Mice were maintained under normal breeding conditions in the Animal Biosafety Level 2 (ABSL2) facility of University of Clermont Auvergne (Clermont-Ferrand, France). All experiments were approved by Comité d’Éthique en Expérimentation Animale C2EA-02 of Clermont-Ferrand (protocol number: CE110-12, CE111-12 and EU0116-3460), follow the guidelines of the Committee for Research and Ethical Issues of the International Association for the Study of Pain (IASP), and in compliance with the ARRIVE relevant guidelines. Stress was induced by NMS for 3 h every day from day 2 after birth until day 14. Wild­type C57BL/6 pups were placed in individual boxes, in a separate room set up with similar environmental conditions. After this period NMS-induced visceral sensitivity was first assessed by performing a colorectal distension (CRD) method described by Larauche et al.^21^. Only mice that showed the highest responses to CRD after NMS were used in this study. After the NMS and CRD procedures, mice were force-fed 200 μL containing 1 × 10^9^ CFU of *B. breve* CNCM I-5644 and/or PBS for 10 days. Each group, the induced-hyperpermeability control group (NMS-PBS) and the induced-hyperpermeability *B. breve* CNCM I-5644 group (NMS-CNCM I-5644), consisted of eight male and six female C57BL/6J mice. The assessment of intestinal permeability (FITC and intestinal tight junction proteins gene expression)^19,63^ and sensibility (colorectal distension [CRD])^51^**,** and Fecal Lipocalin-2 and proteolytic activity^64^ were determinate as before mentioned.

### Statistical analysis

All results were expressed as means ± standard error of the mean (SEM). We performed a one-way ANOVA for normal samples and multiple comparisons were carried out using Tukey’s test. For non-normal samples and/or with unequal variances, non-parametric tests were performed within groups (Kruskal–Wallis test) and multiple comparisons were carried out using Dunn’s test using GraphPad Prism 7 software (GraphPad Software, San Diego, CA, USA). We employed an alpha level of 0.05*.*

## Supplementary Information

Supplementary Information.

## Data Availability

The data generated during this study are available from the corresponding author on reasonable request.
