# Supplementary material for: Serpin-positive Bifidobacterium breve CNCM I-5644 improves intestinal permeability in two models of irritable bowel syndrome
Source: Sci Rep. 2022 Nov 17;12:19776. doi: 10.1038/s41598-022-21746-8 (PMC9672316; doi:10.1038/s41598-022-21746-8)
Supplement: Supplementary file 1 — Supplementary Information. [file 41598_2022_21746_MOESM1_ESM.docx]

**
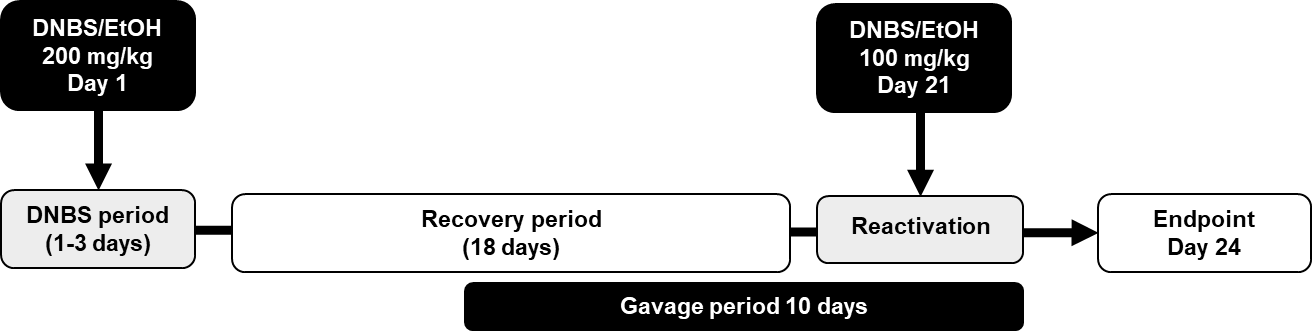
**

**Figure S1**. **DNBS-induced low-grade inflammation experimental protocol**. Colitis was induced by intra-rectal administration of 200 mg/kg of DNBS in solution in 30% ethanol (EtOH). Non-inflamed group received only 30% EtOH in PBS. The effects of DNBS are highest during the first 3 days after its administration (DNBS period). Ten days after the end of the DNBS period bacterial culture or PBS were intra-gastrically administered daily for 10 days (gavage period). Low-grade inflammation was reactivated 21 days after the first DNBS injection with a second injection of 100 mg/kg of DNBS solution. Three days after reactivation mice were sacrificed (Day 24).


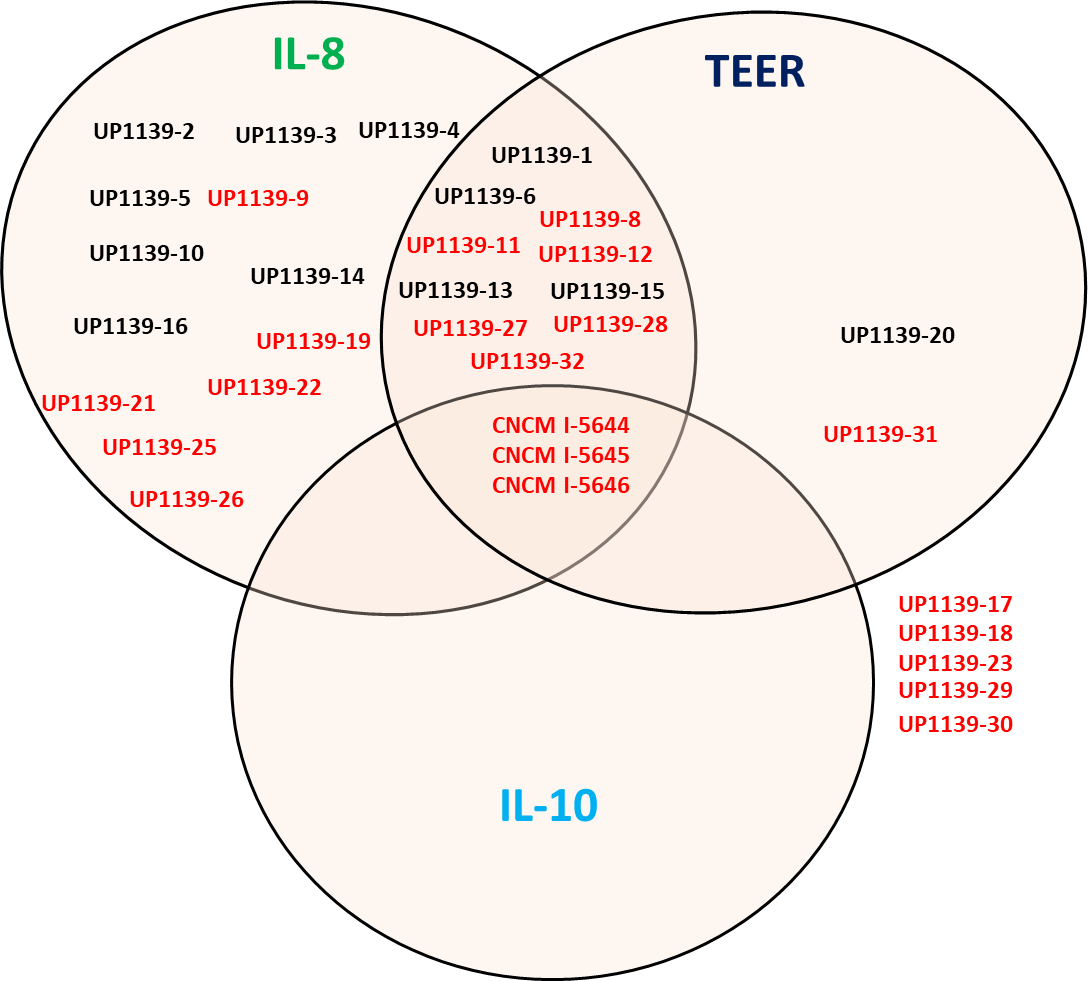


**Figure S2. Venn diagram of selected probiotic properties based on three *in vitro* tests.** i) ability to block TNF*-*α*-*induced interleukin*-*8 secretion from HT-29 cells, ii) to stimulate IL-10 on peripheral blood mononuclear cells (PBMC), and iii) to reinforce the Trans-Epithelial Electrical Resistance (TEER) values in Caco-2 cells. *Bifidobacterium* strains that are PCR positive for serpin are indicated in red .

**Figure S3. Effect of colonic treatment of CNCM I-5644 on sensitivity to colorectal distension (CRD) in neonatal maternal separation (NMS) murine model**. Intracolonic pressure variation (IPV) scores as a function of distention pressure at 20, 40, 60, 80 mmHg (n=16).

**Figure S4. Tight junction intestinal protein regulation in neonatal maternal separation (NMS) model**. Apical junction protein expression levels of *Cingulin* (*Cng*), *Claudin 2* (*Clnd2*), *Occludin* (*Ocln*), and *Tight junction protein 1* (*Tjp1*) were determined by real-time qPCR. The ∆∆CT method was carried out to quantify relative gene expression. The Rpl19 and *Tbp* genes were used as housekeeping genes.

**Table S1. Information on selected intestinal tight junction genes used in this study**.

| Gene symbol | Gene Name | Specie | Assay ID* |
| --- | --- | --- | --- |
| *Ocln* | *Occludin* | Mouse | Mm00500912_m1 |
| *Cldn2* | *Claudin 2* | Mouse | Mm00516703_s1 |
| *Cgn* | *Cingulin* | Mouse | Mm01263534_m1 |
| *Tjp1* | *Tight junction protein 1* | Mouse | Mm00493699_m1 |
| Houskeeping genes | | | |
| *Tbp* | TATA box binding protein | Mouse | Mm01277042_m1 |
| *Rpl19* | Ribosomal protein L19 | Mouse | Mm02601633_g1 |

Quantitative real-time PCR (qPCR) was performed with diluted cDNA (10x) in triplicate and with an iQ5 Real-Time Detection System (Bio-Rad, France, Methods section). The reaction mixture consisted of Sofast Evagreen Supermix (Bio-Rad), primers at 0.5 μM, and 2 μL of diluted cDNA. Values are expressed as relative fold differences normalized to the housekeeping genes, Ribosomal protein L19 (*Rpl19*) and TATA box binding protein (*Tbp*) by the 2-ΔΔCTmethod. All procedures were performed according to the manufacturers’ instructions. *TaqMan Gene Expression Assays (Thermo Fisher Scientific Inc., France)
